# Chimeric Antigen Receptor, Teamwork, Education, Assessment, and Management (CAR-TEAM): A Simulation-Based Inter-professional Education (IPE) Intervention for Management of CAR Toxicities

**DOI:** 10.3389/fonc.2020.01227

**Published:** 2020-08-05

**Authors:** Avis Harden, Dristhi Ragoonanan, Daryl Anildes-Gubman, David McCall, Kathleen Faltus, Sarah Featherston, Basirat Shoberu, Jerelyn R. Moffet, Demetrios Petropoulos, Sajad J. Khazal, Shehla Razvi, Kris M. Mahadeo, Priti Tewari

**Affiliations:** ^1^Department of Pediatrics, University of Texas MD Anderson Cancer Center, Houston, TX, United States; ^2^Teaching, Interprofessional and Simulation Education Center, University of Texas MD Anderson Cancer Center, Houston, TX, United States; ^3^Division of Blood and Marrow Transplant, Department of Pediatrics, Duke Children's Hospital, Duke University, Durham, NC, United States; ^4^Department of Pediatrics, CARTOX Program, The University of Texas MD Anderson Cancer Center, Houston, TX, United States

**Keywords:** inter-professional, education, simulation-based, cytokine release syndrome (CRS), immune effector cell-associated neurotoxicity (ICANS)

## Abstract

Chimeric antigen receptor (CAR) therapies such as tisagenlecleucel, indicated for children and young adults with relapsed and/or refractory CD19^+^ acute lymphoblastic leukemia (ALL), have been associated with striking treatment outcomes and overall survival. Yet, they are also associated with unique and potentially life-threatening complications. Cytokine release syndrome (CRS) and immune effector cell-associated neurotoxicity (ICANS) are generally reversible complications of CAR therapies, but many patients may require critical care support especially if they are not promptly recognized and appropriately managed by frontline healthcare staff. As CAR therapies become more widely available, it is important that inter-professional staff members be aware of general principles regarding diagnosis and management. We hypothesized that an inter-professional education (IPE) simulation-based education intervention (CAR-TEAM) would improve knowledge base and confidence regarding complications of CAR therapies among inter-professional staff. Here, we demonstrate that following CAR-TEAM training, >90% of participants demonstrated knowledge proficiency and confidence in the IPE content area. CAR-TEAM training may serve as an important tool to establish initial and continued competency among sites introducing CAR therapies.

## Introduction

Cancer immunotherapies have been associated with remarkable response rates but are also associated with unique and potentially severe toxicities, which can lead to rapid and life-threatening cardiorespiratory and/or neurological clinical deterioration. In 2017, an autologous chimeric antigen receptor (CAR) T cell therapy (tisagenlecleucel) indicated for children and young adults with relapsed and/or refractory CD19^+^ acute lymphoblastic leukemia (ALL) became the first gene therapy to be approved in the USA (1). CAR T cell therapies are generated through genetic modification of the patient's own (autologous) T cells or those of an allogeneic donor. The isolated cells are activated and genetically modified via viral transduction or non-viral gene transfer, to express an engineered chimeric cell-surface receptor comprising an extracellular antigen-recognition domain; this is usually an antibody single-chain variable fragment (scFv), linked to at least one intracellular signaling domain—usually the CD3ζ chain of the T cell receptor plus one or more domains derived from co-stimulatory receptors, such as CD28 or 4-1BB ligand receptor (4-1BB; also known as TNFRSF9) (2, 3). The extracellular portion of the CAR enables recognition of a specific antigen (such as CD19), and the signaling domains stimulate T cell proliferation, cytolysis, and cytokine secretion to enable elimination of the target (4–6).

Tisagenlecleucel has been associated with ~76% overall survival at 12 months among patients with relapsed/refractory disease who previously had no curative options (7). Yet, unique toxicities such as cytokine release syndrome (CRS) and immune effector cell-associated neurotoxicity (ICANS) are potentially life-threatening complications of this therapy. The pathophysiological mechanisms of both CRS and ICANS remain poorly understood (8). CRS typically presents as a systemic inflammatory response associated with CAR cell proliferation, involving immune response-modulating proteins and cytokines, characterized by fever, hypoxia, tachycardia, hypotension, and multi-organ dysfunction (FDA)^1^ (9). ICANS can occur concurrently with CRS, following its resolution, or without associated CRS and is characterized by encephalopathy, delirium, seizures, and, at times, cerebral edema. Some patients who receive tisagenlecleucel may require intensive monitoring and critical care support, predominantly owing to these toxicities, especially if they are not promptly recognized and appropriately managed by frontline healthcare staff (6, 10).

We have previously collaborated with the Hematopoietic Cell Transplantation-Cancer Immunotherapy (HCT-CI) Subgroup of the Pediatric Acute Lung Injury and Sepsis Investigators (PALISI) Network and the Pediatric Transplantation and Cellular Therapy Consortium (PTCTC) to develop guidelines for the grading and management of CRS and ICANS in pediatric patients (6), and key components were subsequently adopted by the American Society of Transplantation and Cellular Therapies (ASTCT) in their proposed consensus grading system (8).

As CAR therapies expand from select medical centers and become more widely available, it is essential that treating facilities and their local partners (who may, for example, see unanticipated patients in their local emergency rooms) have adequate clinical infrastructure in place. Inter-disciplinary staff should be appropriately trained to promptly recognize and manage complications of CAR therapies. While specific management algorithms may vary based on institutional preference, CAR product, and/or patient population, there are overall guiding principles that may be considered, as shown in Table 1 (6, 8). In 2019, we collaborated with the Association for Pediatric Hematology Oncology Nursing (APHON) and PALISI-HCT-CI to develop an inter-professional education (IPE) didactic and simulation (SIM) training intervention. We hypothesized that our interdisciplinary training intervention (CAR-TEAM) would improve knowledge base and confidence regarding complications of CAR therapies among interdisciplinary staff. We aimed for >90% of participants to demonstrate knowledge proficiency and confidence in the IPE content area. If successful, this could serve as an important tool to establish competency among sites introducing CAR therapies.

**Table 1:** ASTCT CRS and ICANS Consensus Grading and General Management Principles (8).

**ASTCT CRS consensus grading**					**General management**
**CRS parameter**	**Grade 1**	**Grade 2**	**Grade 3**	**Grade 4**	
Fever	Yes	Yes	Yes	Yes	Supportive care with antipyretics, broad-spectrum antibiotics if neutropenic, hydration, seizure prophylaxis, IL-6 antagonists for fever lasting greater than 3 days
		**With**			
Hypotension	No	Requiring IV fluids but not requiring vasopressors	Requiring one vasopressor with or without vasopressin	Requiring multiple vasopressors (excluding vasopressin)	Fluid boluses, IV hydration, IL-6 antagonists, corticosteroids, vasopressors depending on grade
		**And/Or**			
Hypoxia	No	Requiring low-flow O_2_ via nasal cannula^3^ or blow-by	Requiring O_2_ via high-flow nasal cannula, facemask, non-rebreather mask, or Venturi mask	Requiring O_2_ via positive pressure (e.g., CPAP, BiPAP, and mechanical ventilation)	Supplemental oxygen, respiratory support, IL-6 antagonists, corticosteroids depending on grade
**ICANS**	**Grade 1**	**Grade 2**	**Grade 3**	**Grade 4**	**Management**
Neurotoxicity Domain	ICE score for children age ≥12 years: 7–9 CAPD score for children <12 years: 1–8 Encephalopathy and/or depressed level of consciousness	ICE score for children age ≥12 years: 3–6 CAPD score for children <12 years: 1–8 Encephalopathy and/or depressed level of consciousness	ICE score for children age ≥12 years: 0–2 CAPD score for children <12 years: ≥9 Encephalopathy and/or depressed level of consciousness Seizure Focal cerebral edema	Encephalopathy and/or depressed level of consciousness Seizure Motor weakness Diffuse cerebral edema or raised intracranial pressure	Supportive care with IV hydration, switch to IV medications and nutrition to prevent aspiration, avoidance of central nervous system depressants. Use of anti-epileptics for seizure management, IL-6 antagonists, and corticosteroids if ICANS is associated with CRS

## Methods

This retrospective study was approved by the institutional review board at the University of Texas at MD Anderson Cancer Center. We developed an IPE module in collaboration with content experts (faculty physicians, clinical fellows, pharmacists, and nursing from cellular therapy, and critical care units) from the MD Anderson CARTOX Program, APHON and PALISI, to address evidence-based management for CAR therapy-related toxicities in pediatric patients. We aimed to increase awareness of the risk factors for and improve recognition of CRS and ICANS and foster confidence in general management strategies for these complications. We hypothesized that as centers introduce CAR therapies at their centers and on-board new staff, simulation training may create a safe environment to learn and promote team confidence. If successful, teams could consider similar training exercises at their home centers.

The module included a pre-intervention knowledge and confidence assessment and didactic session followed by an IPE simulation exercise. The didactic lecture included information on indications for CAR therapy in pediatrics, clinical candidate selection, an overview of product collection and manufacture, infusion reactions and post-CART complications (including ASTCT grading and the Cornell Assessment of Pediatric Delirium—CAPD tool), along with general management principles (8, 11). Participants were also educated on the use of the SBAR (situation, background, assessment, and recommendation) communication tool to help facilitate improved communication and hand over quality between team members (12). Emphasis was placed on team dynamics and communication as well as appropriate escalation of care. Post-intervention knowledge and confidence assessments were performed immediately after and at 90 days. Our primary objective was to determine whether >90% of participants (i) either “agreed” or “strongly agreed” that they felt confident in specified CAR therapy diagnosis and management areas and (ii) demonstrated knowledge proficiency as determined by accurate responses to written questions.

MD Anderson's CARTOX Program provides oversight for the care for the hospital's CAR therapy patients and is the first stand-alone immune effector cellular therapy program to earn accreditation from the Foundation for the Accreditation of Cellular Therapy (FACT). The PALISI Network is a national organization devoted to identifying therapeutic and preventative strategies for acute respiratory distress syndrome, sepsis, multi-organ failure, and other acute life-threatening pulmonary or systemic inflammatory syndromes that affect infants and children through multi-center research. The Association of Pediatric Hematology/Oncology Nurses (APHON) is a professional organization for pediatric hematology/oncology nurses and other pediatric hematology/oncology healthcare professionals dedicated to promoting optimal nursing care for children, adolescents, and young adults with cancer and blood disorders.

IPE Module: Pre- and post-intervention confidence assessments were developed and reviewed by three content and/or education experts for content validity. A subject content expert led a didactic lecture on CRS and ICANS with course content reviewed and approved by the IPE committee. Inter-professional learners were then selected in small groups of 5–6 people to participate in a simulated exercise of a pediatric patient receiving CAR therapy who progressed through the various grades (per ASTCT criteria) of CRS and ICANS. These grades were separated into three phases of the simulated exercise. The goals of phase I and II were to recognize and effectively manage grades 1 and 2 CRS and grades 3 and 4 CRS, respectively. Phase 3 was aimed at recognition and management of ICANS. A patient caregiver, vital signs monitor, emergency equipment, medication dosing sheet, and a telephone to place a consult/escalate care were also provided. Participants were encouraged to approach the simulation from the perspective of their respective field (cellular therapy vs. critical care, physician, nurse etc.), but above all, to focus on teamwork and optimal communication. At the end of each phase, participants were asked to give a hand-off sheet summarizing the patient's clinical background, current clinical status, and, when appropriate, requirements for escalation of care. An objective checklist was used during each phase to ensure that key goals were achieved including the recognition and accurate grading of CRS and/or ICANS, appropriate general management based on CRS and/or ICANS grade, escalation of care when necessary, and use of effective and closed loop communication among team members as detailed in Table 2. All participants were then debriefed by a panel of experts from education, cellular therapy, critical care, renal, pharmacy, and nursing to identify further learning opportunities at the end of each phase. A post-IPE and simulation survey was distributed to assess participants' confidence of their knowledge base as well as assess general competency. Questions probed the participant's ability to appropriately identify risk factors and signs and symptoms of CRS and ICANS; general management principles were also assessed. Participants were encouraged to learn their institution-specific management algorithms as these may vary based on center.

**Table 2:** Outline of simulation scenarios.

**Objective assessment of expected interventions**
**Phase I: recognition and management of CRS grades 1 and 2**
• Initial clinician recognized early signs of CRS • Patient's change in clinical status appropriately communicated to team members and care escalated • Provider recognized and appropriately graded CRS based on clinical scenario • Appropriate implementation of management guidelines for CRS grades 1 and 2
**PAUSE SIMULATION AND DEBRIEF**
**Phase II: recognition and management of CRS grades 3 and 4**
• Recognition of progression of CRS to grades 3 and 4 • Patient's change in clinical status appropriately communicated to team members and care escalated • Appropriate clinical assessment performed by team • Appropriate implementation of management guidelines CRS grades 3 and 4 including consideration of appropriate laboratory and imaging tests, initiation of vasopressors for hypotension unresponsive to fluid boluses, use of supplemental oxygen, steroids, and IL-6 antagonist if not administered previously • Escalation of care and involvement of the pediatric intensive care team • Utilization of the communication tool such as SBAR and closed-loop communication to improve team function and allow effective transfer of patient care
**PAUSE SIMULATION AND DEBRIEF**
**Phase III: recognition and management of ICANS**
• Changes in patient's mood/behavior recognized as an early sign of ICANS • Patient findings appropriately communicated to team members and care escalated • Appropriate clinical assessment performed by team including CAPD assessment and neurological examination • Progression of ICANS recognized throughout scenario as patient's clinical status progressively declines • Appropriate implementation of management guidelines for ICANS including consideration of appropriate laboratory and imaging tests and seizure management • Consideration of relevant consultation with other specialties as needed with effective communication through the utilization of the SBAR communication tool
**PAUSE SIMULATION AND DEBRIEF**
• CRS: Cytokine release syndrome • SBAR: Situation, background, assessment, and recommendation • ICANS: Immune effector cell-associated neurotoxicity

Statistical Analysis: Descriptive statistics was used to summarize participants' demographics and baseline characteristics.

## Results

As shown in Table 3, participants (*n* = 70) represented diverse centers and disciplines from across the United States, including oncology, hematology, and critical care.

**Table 3:** Characteristics of CAR-TEAM participants.

**Characteristic**		**Participants *n* = 70 (%)**
Profession	Staff nurse	29 (41.4%)
	Nurse practitioner	5 (7.1%)
	Educator	3 (4.3%)
	Nurse manager	3 (4.3%)
	Clinical nurse specialist	3 (4.3%)
	Clinical nurse coordinator	2 (2.9%)
	Physician assistant	4 (5.7%)
	Fellow	10 (14.3%)
	Physician	10 (14.3%)
	Pharmacist	1 (1.4%)
Areas of specialty	Academics	1 (1.4%)
	Administration/management	1 (1.4%)
	Ambulatory	1 (1.4%)
	Biotherapy	2 (2.9%)
	Critical care	12 (17.1%)
	Hematology	16 (22.9%)
	Leukemia/lymphoma	10 (14.3%)
	Oncology	25 (35.7%)
	Neuro-oncology	4 (5.7%)
	Pain management	2 (2.9%)
	Sickle cell	5 (7.1%)
	Solid tumors	7 (10.0%)
	Staff education	5 (7.1%)
	Surgical oncology	5 (7.1%)
	Symptom management	2 (2.9%)
	Unknown	25 (35.6%)
Practice settings	Hospital (Inpatient)	40 (57.1%)
	Hospital (Outpatient)	6 (8.6%)
	School of nursing	1 (1.4%)
	Unknown	23 (32.9%)
Average number of patients seen in your clinical practice per week	0–20	35 (50%)
	20–40	7 (10%)
	40–60	2 (2.9%)
	>60	1 (1.4%)
	Unknown	25 (35.7%)
No. of years of experience	<5	7 (10.0%)
	5–10	20 (28.6%)
	11–20	11 (15.7)
	>20	7 (10.0%)
	Unknown	25 (35.7%)

### Inter-professional Confidence Assessment

**Figure 1:**
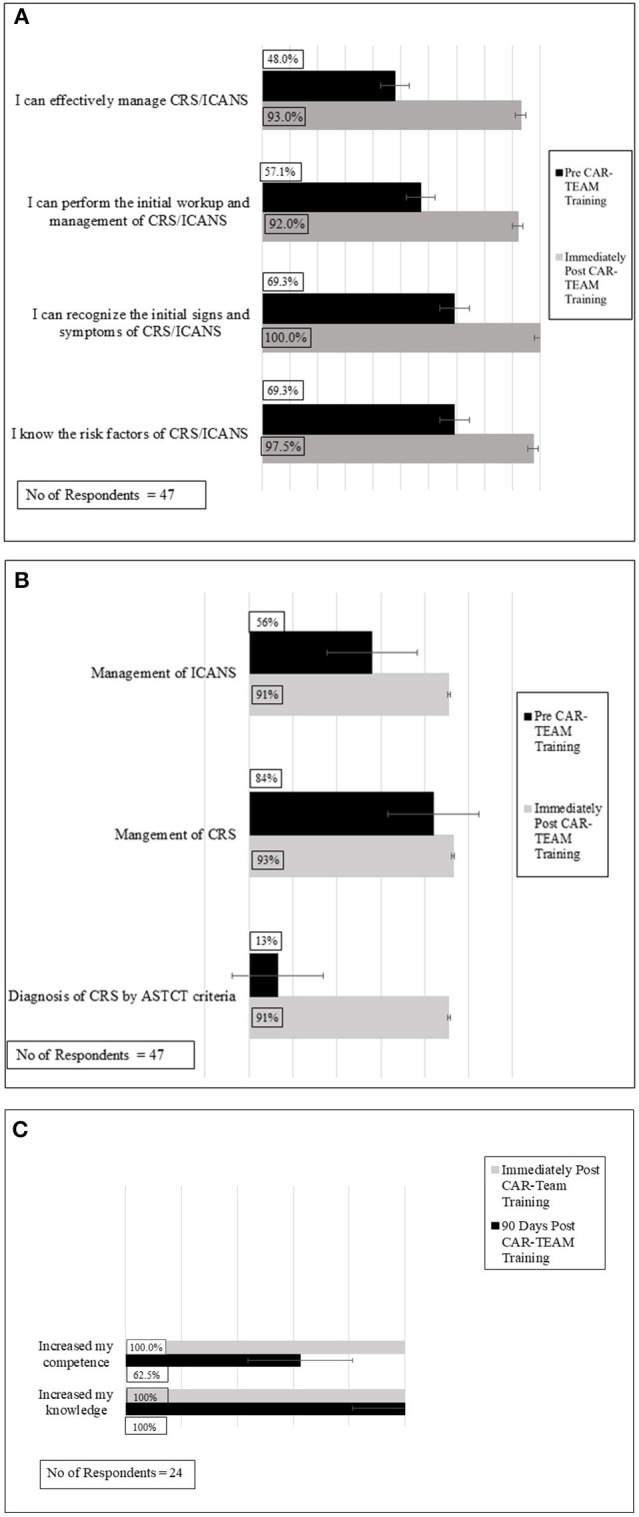
Confidence and knowledge assessments of participants pre and immediately post-CAR-TEAM Training: significant improvements were seen in both confidence **(A)** and knowledge base **(B)** following CAR-TEAM training. **(C)** Overall evaluation of participants immediately post-CAR-TEAM training and 90 day post- post-CAR-TEAM training.

As shown in Figure 1A, pre- and post-IPE assessments indicate significant improvement in the confidence of the 47 participants (66% survey response rate) who responded to the post-assessment survey regarding (i) knowledge of risk factors for CRS and/or ICANS, (ii) recognition of initial signs/symptoms of CRS and/or ICANS, (iii) ability to conduct initial work-up of CRS and/or ICANS, and (iv) effective management of CRS and/or ICANS. In all areas assessed, >90% of the 47 respondents expressed confidence post-IPE intervention.

### Inter-professional Knowledge Assessment

Figure 1B shows significant improvements in knowledge assessments pre- and post-IPE intervention. Specifically, post SIM >90% of the 47 participants who responded to the survey demonstrated knowledge proficiency regarding (i) indications for admission to the intensive care unit, (ii) diagnosis of CRS by ASTCT criteria, (iii) management of CRS, and (iv) management of ICANS.

### Immediate Overall Post-IPE Evaluation

Immediately following completion of the IPE intervention, participants were surveyed regarding their overall perceptions. Of the 47 respondents, all 47 respondents “agreed” or “strongly agreed” that they felt more confident in (i) their patient management, (ii) their knowledge base, and (iii) their clinical performance following the IPE intervention (Figure 1C).

### 90-Day Overall Post-IPE Evaluation

To assess durability of the IPE intervention, voluntary surveys were sent to participants by APHON, 90 days after the IPE intervention. The APHON survey consisted of three questions sent via electronic mail. There was a 34% response rate (*n* = 24). Of the 24 respondents, all respondents agreed that the IPE intervention had increased their knowledge (Figure 1C). When asked if they felt that their clinical practice had improved as a result of the IPE intervention, 22 of the 24 respondents of survey participants agreed. Further, 22 survey respondents agreed that that they were able to apply new and relevant information to their practice as a result of the IPE intervention. Overall, participants felt that the workshop helped improve clinical practice, increased knowledge, and improved the ability to apply the acquired knowledge to clinical practice.

## Discussion

As health institutions increasingly provide access to CAR therapies, adequate strategic and operational planning and preparation are needed to ensure the safe delivery of such treatments (6). CRS and ICANS are the most common toxicities with CAR therapies occurring in 60–94 and 70–80% of patients, respectively. While both syndromes are reversible, they can be severe and life-threatening if left untreated. Prompt and appropriate intervention is key (13). Immune effector cell (IEC) center accreditation by the FACT is a voluntary means of ensuring adherence to quality standards; it requires, among other components, initial education and ongoing competency training for inter-professional staff directly involved in the care of CAR therapy patients. This includes but is not limited to cellular therapy, critical care, triage and neurology, physicians, trainees, nurses, pharmacists, advance practice providers and medical assistants, and research and data personnel (6, 14). Additionally, emergency medical services, community hospitals, and local urgent care facilities may require high vigilance to recognize and promptly escalate care in the event that a patient treated with CAR therapy presents to their facility in an emergency (6).

IPE cultivates collaborative practice to provide patient-centered healthcare (15, 16). IPE programs are increasingly recognized as an important tool to reduce medical errors and improve communication (17, 18). Simulation-based training is a safe and effective means of educating inter-professional care providers (from novice to experienced levels) in various clinical settings, from surgical to critical care management in technical abilities and/or effective and practical non-technical skills such as problem-solving (19, 20). High-fidelity mannequin-based simulations provide training for smaller groups and there is evidence that technology-enhanced simulation can provide comparable results and may be useful for a larger number of trainees (21). Simulation-based training modules (SIM), such as those established by the American Heart and Lung Association for life support training, afford providers the opportunity to safely manage these challenging complications by providing lifelike clinical education opportunities (21–23). SIM training can provide real-time clinical education and subsequently bolster provider confidence without risking patient well-being (23). Most importantly, SIMs also allow for the practice of inter-professional provider communication and foster a sense of teamwork in a safe setting (24, 25). Further, computer-based virtual reality simulators may be accessible either on-site at an institution or via personal devices that can be accessed at a participant's leisure. This type of simulation education has been used successfully for years in adult, pediatric, and neonatal resuscitation courses (21).

Our IPE intervention facilitated critical care and cellular therapy providers from across the United States to learn jointly, which they may not otherwise have had the ability to do, as they likely attend more discipline-specific meetings. Positive overall confidence self-assessments suggest that the IPE intervention was not limited by fear of training in an inter-professional setting and/or in the presence of learners at different experience levels. Knowledge proficiency assessments suggest that the IPE intervention was effective in training learners regarding CAR therapy management.

Our study may have been limited by the survey response rates. The immediate post-assessments were completed electronically and required participants to use their smartphones. Not all participants were able to successfully access this survey system. Further, responder bias could have influenced our conclusions, in particular with our long-term follow-up surveys. As survey responses may sometimes be skewed by those who had strongly positive and/or negative perceptions, we find it encouraging that we did not receive any overwhelmingly negative responses. We expect the real-world value of our IPE to be at least satisfactory for consideration at individual centers. We did not intend to assess whether our IPE resulted in improved patient outcomes, but this can be assessed at individual centers in prospective studies. To increase survey responses in the future, the use of electronic tablets for those unable to access the survey on their smartphone at the education site, follow-up reminders to complete surveys via email, telephone surveys, or incentives may have improved the response rate if utilized.

Our institution has established integrated simulation training for CAR therapy toxicity recognition and management into a pediatric foundational orientation for all newly hired employees involved in the care of patients receiving IEC therapy. This simulation training is also being expanded to the adult patient care departments. We are currently exploring development of an internet-based simulation training that will augment our live module and facilitate larger-scale outreach.

## Data Availability Statement

All datasets presented in this study are included in the article/supplementary material.

## Author Contributions

KM conceptualized the project and obtained grant funding. AH, DR, DA-G, DM, KF, BS, JM, SF, SR, KM, and PT developed the IPE module. AH, DR, KM, and PT wrote the manuscript. All authors reviewed and/or edited the manuscript and made meaningful contributions before submission.

## Conflict of Interest

KM has received an unrestricted medical education grant from Jazz Pharmaceuticals. The remaining authors declare that the research was conducted in the absence of any commercial or financial relationships that could be construed as a potential conflict of interest.

## Footnotes

^1^FDA Briefing Document: Oncologic Drugs Advisory Committee meeting; BLA 125646 Tisagenlecleucel. Available online at: https://www.fda.gov/media/106081/download (accessed 2020).
